# Editorial: Hypocretins/Orexins

**DOI:** 10.3389/fendo.2020.00357

**Published:** 2020-06-02

**Authors:** Miguel López, Luis de Lecea, Carlos Diéguez

**Affiliations:** ^1^Department of Physiology, CIMUS, Instituto de Investigación Sanitaria de Santiago de Compostela (IDIS), Santiago de Compostela, Spain; ^2^CIBER Fisiopatología de la Obesidad y Nutrición, Santiago de Compostela, Spain; ^3^Department of Psychiatry and Behavioral Sciences, Stanford University School of Medicine, Stanford, CA, United States

**Keywords:** hypocretin, orexin, energy balance, fear, plasma, cancer

Independently discovered in 1998 by the groups of de *Lecea & Sutcliffe* and *Sakurai & Yanagisawa*, hypocretins (HCRT), also known as orexins (OX), are two hypothalamic neuropeptides derived from the same precursor mainly expressed in the peripherical (Pef) and lateral (LHA) hypothalamic areas (1, 2). Two different HCRT/OX receptors have been described, named hypocretin/orexin 1 receptor (HCRTR1/OX1R) and hypocretin/orexin 2 receptor (HCRTR2/OX2R) (1, 2). Although they were initially described to be involved in feeding regulation, HCRT/OX have pleiotropic activities that regulate homeostatic processes, such as feeding, sleep, endocrine and cardiovascular function, and thermogenesis, as well as arousal, reward, and mood (3–6). In fact, HCRT/OX play important roles in a series of human diseases, including narcolepsy, obesity, and drug addiction (3–6).

The present Research Topic includes 2 review articles and 3 original papers. These articles revisit some of the most classical aspects of HCRT/OX and provide new insights into the field, such as their role in tumorogenesis and fear. This assembly of work from experts in the topic make clear the growing data implicating HCRT/OX in physiology and disease and the possibilities of developing drugs targeting their signaling pathways.

## Original Research Articles

Dustrude et al. studied the effect of HCRT/OX neurons in the PeF, which send projections to the amygdala, a critical region involved in fear learning and fear expression. Using electrophysiology, pharmacology, and chemogenetic approaches, they show that HCRT/OX activates medial neurons of the central nucleus of the amygdala (CeA), through HCRTR1/OX1R (but not HCRTR2/OX2R), leading to modified presynaptic release of glutamate inside this area. Importantly, both systemic and specific intra-amygdala administration of a HCRTR1/OX1R (but not HCRTR2/OX2R) antagonist, namely compound 56, reduced the expression of conditioned fear. This evidence suggests that the PeF-CeA HCRT/OX neuronal pathway modulates fear and that the selective HCRTR1/OX1R antagonist could be a treatment for fear-related disorders.

In their original paper Mäkelä et al. reanalyzed the plasma concentration of HCRT1/OX-A using a novel specific and sensitive OX-A radioimmunoassay (RIA) with a solid phase extraction method and using an OX-A antibody raised in rabbits. Their data show that plasma HCRT1/OX-A concentrations are very variable in human subjects (range 0.5–16 pg/ml) and notably, they do not correlate with feeding or wake/sleep cycle, quite opposite to other control hormones assayed, such as cortisol and melatonin. Overall, these data suggest that HCRT1/OX-A is present in low amounts in blood and does not follow a circadian pattern. This indicates that correlative studies between circulating HCRT1/OX-A and other physiological parameters under circadian control, such as metabolism and/or sleep/wake cycle must be carefully deciphered.

Suo et al. investigated the effect of HCRT1/OX-A in tumorogenesis, more specifically on cell proliferation in pancreatic PANC1 cancer cells. Their results show that activation of HCRTR1/OX1R or treatment with HCRT1/OX-A promotes cell proliferation in PANC1 cells through inhibiting cell apoptosis. The molecular mechanism by which HCRT1/OX-A exerts these actions involves the activation of the AKT/mTOR (protein kinase B/mechanistic target of rapamycin) signaling pathway, leading to cell proliferation by inhibiting Bcl-2/caspase-9/c-myc-mediated apoptosis. These *in vitro* findings might suggest that activation of HCRTR1/OX1R could be important in the development of pancreatic cancer and that this receptor could be a potential therapeutic target.

## Reviews

In direct line with the aforementioned original paper, Couvineau et al. review the antitumoral effect of HCRT/OX mediated by a new signaling pathway involving the presence of two immunoreceptor tyrosine-based inhibitory motifs (ITIM) in both HCRT/OX receptor subtypes. They speculate that, based on this new mechanism, the HCRT/OX signaling might be a possible therapeutic target in several types of cancer, such as colonic, pancreatic, prostatic, adrenocortical, and hepatocellular carcinoma.

Finally, Milbank and López revisited the initially descried actions of hypothalamic HCRT/OX on energy homeostasis. They focus on the interaction between the HCRT/OX system and novel cellular pathways involved in the central control of metabolism, such as the energy sensor AMP-activated protein kinase (AMPK) and also proteostatic mechanisms, such as unfolded protein response (UPR) and endoplasmic reticulum (ER) stress.

## Conclusions

The objective of this Research Topic was to celebrate the 20th anniversary of the discovery of HCRT/OX by revisiting classical and new knowledge of this family of neuropeptides. Taken together, this compilation demonstrates the diverse molecular and physiological functions of HCRT/OX, their role in pathology and their potential as therapeutic targets.

## Author Contributions

All authors listed have made a substantial, direct and intellectual contribution to the work, and approved it for publication.

## Conflict of Interest

The authors declare that the research was conducted in the absence of any commercial or financial relationships that could be construed as a potential conflict of interest.
